# Physiological and Biochemical Responses of *Stylosanthes* spp. Under Water Deficit Conditions

**DOI:** 10.3390/plants15121819

**Published:** 2026-06-12

**Authors:** Vitor Oliveira dos Santos, Marilza Neves do Nascimento, Daniel Lucas Santos Dias, Robson de Jesus Santos, Uasley Caldas de Oliveira, Aritana Alves da Silva, Lorena Passos de Souza, Claudineia Regina Pelacani

**Affiliations:** Department of Biological Sciences, State University of Feira de Santana, Feira de Santana 44036-900, BA, Brazil; mnnascimento@uefs.br (M.N.d.N.);

**Keywords:** forage legume, stress physiology, estilosantes, tolerance, semiarid

## Abstract

Studies aimed at identifying genotypes tolerant to water deficit are essential for the development of superior plant materials adapted to regions with limited water availability, such as the Brazilian Semi-Arid. This study evaluated the physiological, biochemical, and enzymatic responses of *Stylosanthes* spp. subjected to different levels of water availability (60%, 40%, and 20% of pot capacity). The experiment was conducted using a completely randomized design using a 3 × 2 factorial scheme, comparing the accession BGF 11-001 and the cultivar BRS-Bela (cv. Bela). Physiological traits, biochemical variables, and antioxidant enzyme activity were analyzed. The accession BGF 11-001 showed resilience under water deficit, maintaining high chlorophyll content even under severe stress. This response was associated with increased accumulation of amino acids such as proline, as well as enhanced antioxidant activity, indicating a tolerance mechanism based on osmotic adjustment and cellular protection. In contrast, cv. Bela exhibited higher sensitivity to water stress, with a pronounced reduction in photosynthetic pigments and greater accumulation of compatible solutes, including total soluble proteins, reducing sugars, amino acids, and proline, without significant activation of antioxidant enzymes. Overall, the results demonstrate that the genotypes adopt distinct strategies to cope with water stress, with BGF 11-001 being more efficient in activating defense mechanisms. Therefore, BGF 11-001 has agronomic potential for cultivation in drought-prone regions and is a promising genetic resource for forage breeding programs aimed at improving drought tolerance.

## 1. Introduction

The Brazilian Semiarid region corresponds to approximately 12% of the national territory and is mainly concentrated in the northeastern region of the country [1]. This region is characterized by recurrent periods of negative water balance resulting from irregular rainfall distribution throughout the year [2,3]. In light of these adverse climatic conditions and the limited availability of drought-adapted genotypes, agricultural activity in the region faces serious challenges, compromising local socioeconomic development. Non-drought-tolerant crop plants experience a marked reduction in productivity under limited water availability. In forage species, this directly affects biomass production and consequently reduces the availability of feed for grazing.

The response of plants to water deficit is a complex process that is highly dependent on the species and genotype [4], given that reduced water availability can trigger physiological and biochemical changes that enable tolerance to this abiotic condition [5,6,7]. These changes include the decrease in leaf water potential [8], reduction in photosynthetic activity, accumulation of compatible solutes, and the increase in the activity of enzymatic and non-enzymatic antioxidant mechanisms that mitigate oxidative stress by reducing the concentration of reactive oxygen species (ROS) in plant tissues [4,5,7,9,10,11].

Comparative studies between drought-tolerant and drought-sensitive genotypes have shown that drought tolerance is closely associated with coordinated metabolic adjustments under water deficit conditions [12,13,14]. In wheat, drought-tolerant genotypes exhibit greater metabolic stability and accumulate higher levels of compatible solutes, such as proline, soluble sugars, and organic acids, under water stress compared with sensitive genotypes [12]. In clusterbean (*Cyamopsis tetragonoloba* (L.) Taub.) transcriptomic studies have demonstrated that drought tolerance is associated with the induction of genes related to catalytic activity, energy metabolism, and photosynthesis, including pathways such as transketolase, phosphoenolpyruvate carboxylase, and cytochrome oxidase, whereas drought sensitivity is characterized by the repression of genes encoding antioxidant enzymes [13]. These differences indicate that tolerant plants maintain a greater capacity for metabolic adjustment and redox balance regulation, thereby supporting cellular homeostasis under water deficit conditions. In contrast, sensitive plants show greater functional impairment owing to their lower efficiency in detoxifying reactive oxygen species [13,14].

The genus *Stylosanthes* Sw. (Fabaceae Lindl.) is predominantly composed of perennial species and is recognized as a plant genetic resource owing to its importance in forage production [15] and is widely used in the establishment of high-quality pastures. This prominence is largely due to the nutritional composition of these species, with protein content ranging between 12% and 20% [16]. Moreover, drought tolerance presented by the genus [15,16,17,18] is a highly relevant trait for selecting genetic materials adapted to environments with climatic adversities, such as the Brazilian Semiarid region.

Ferreira-Neto et al. [19] observed that *Stylosanthes scabra* Vogel. rapidly mobilizes biochemical strategies when subjected to water deficit, particularly through the accumulation of proline, which contributes to reducing cellular osmotic potential and promotes water influx and retention in plant tissues—an important mechanism of osmotic adjustment under stress conditions. Han et al. [20] reported a significant increase in catalase activity in *Stylosanthes guianensis* (Aubl.) Sw. under water stress. The enhanced activity of this enzyme indicates its crucial role in the decomposition of hydrogen peroxide (H_2_O_2_), a highly harmful reactive oxygen species that can damage cellular structures, especially DNA and membranes, thus contributing to the maintenance of redox homeostasis during stress [20].

The Forage Germplasm Bank of the State University of Feira de Santana (BGF-UEFS) conserves part of the genetic variability of the *Stylosanthes* genus present in the Brazilian Semiarid, originating from collections carried out during expeditions in the semiarid region of Bahia [21]. Studies such as those by Oliveira et al. [22] have already demonstrated morphological variability among the accessions maintained in this bank. However, more in-depth studies are needed to elucidate the physiological and biochemical mechanisms associated with drought tolerance in these genotypes. Investigations of this nature are essential for the development of forage cultivars adapted to arid and semi-arid regions, where water availability is frequently limited.

Given the need for forage genetic materials adapted to the climatic constraints of the Brazilian Semiarid region and considering the limited physiological and biochemical information available for *Stylosanthes* accessions maintained in the BGF-UEFS collection, this study aimed to characterize their responses to water deficit. By integrating physiological, biochemical, and multivariate analyses, we provide novel insights into the mechanisms underlying drought tolerance in *Stylosanthes* spp., thereby contributing to the identification of promising genetic materials for use in semi-arid environments.

## 2. Results

The interaction between factors was significant for Chl*a* (*p* ≤ 0.01), total chlorophyll (Chl*tot*) (*p* ≤ 0.05), and Ψw (*p* ≤ 0.05), indicating that the genotypes’ responses to water conditions varied according to the levels of these factors. The genotype factor had a significant effect on Chl*b* (*p* ≤ 0.01), showing variation in the concentration of this pigment among the genotypes evaluated. In turn, water availability had a significant effect on WUE (*p* ≤ 0.05) and Chl*b* (*p* ≤ 0.01), highlighting that this factor plays an important role in water use efficiency under different water supply conditions.

**Figure 1:**
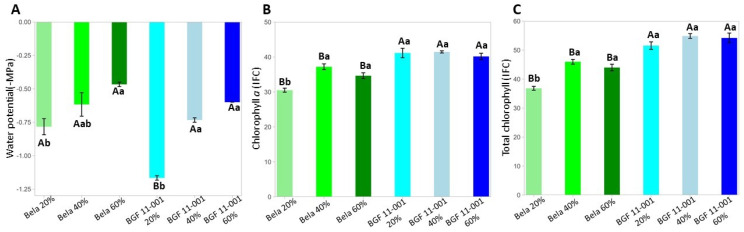
Breakdown of the interaction of Water Potential (**A**), Chlorophyll *a* (**B**), and Total Chlorophyll (**C**) in *Stylosanthes* spp. grown under different water availability conditions. Means followed by different letters (uppercase for the genotype factor and lowercase for the water availability factor) differ significantly according to Tukey’s test (*p* ≤ 0.05). Error bars indicate the standard deviation of the mean.

The breakdown of the interaction for Ψw (Figure 1A) showed variation between genotypes only at 20% pot capacity. Under these conditions, cv. Bela maintained a significantly higher water potential compared to BGF 11-001. When analyzing water availability within each genotype, BGF 11-001 showed similar means for the 40% and 60% levels, but significantly different from the 20% level. However, cv. Bela showed a significant difference only between the 20% and 60% levels, with the 40% level being statistically similar to both.

The contents of Chl*a* (Figure 1B) and Chl*tot* (Figure 1C) exhibited similar behavior regarding the interaction between factors, and for both variables BGF 11-001 showed higher values under the two water availability conditions. Additionally, when evaluating genotype performance according to water availability, cv. Bela showed a significant decrease under severe deficit (20%) compared to the other levels.

**Figure 2:**
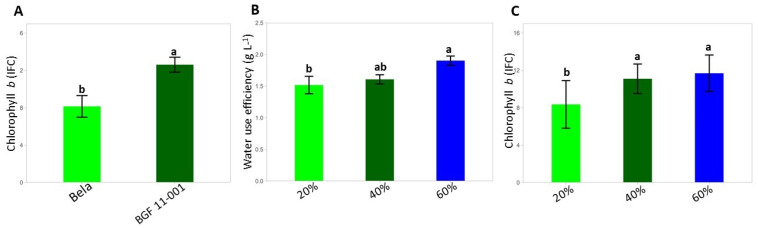
Mean chlorophyll *b* content as a function of the individual effects of genotype (**A**) and water availability (**C**), as well as water-use efficiency assessed as a function of water availability (**B**) in *Stylosanthes* spp. grown under different soil moisture levels. Different letters indicate significant differences according to Tukey’s test (*p* ≤ 0.05). Error bars represent standard deviation of the mean.

In contrast, the BGF 11-001 accession maintained stable performance for these variables, regardless of the water conditions applied in this study. In the individual analysis of the genotype factor for Chl*b* content (Figure 2A), it was observed that the BGF 11-001 accession had significantly higher values than cv. Bela. The comparison of means for the individual effect of water availability revealed significant differences in WUE (Figure 2B) for both genotypes evaluated, especially between the 20% and 60% availability levels. Although this difference was observed between these two levels, the treatment under moderate deficit (40%) did not differ significantly from the others. Regarding Chl*b* content (Figure 2C), a reduction was observed only under severe water deficit conditions (20%).

For the biochemical variables, the interaction between genotype and water availability was significant for total soluble proteins (TSP) (*p* ≤ 0.01) and reducing sugars (RS) (*p* ≤ 0.05), suggesting that the responses of the genotypes to different levels of water availability varied depending on the combination of these factors. However, for amino acids (AAs), the interaction was not significant, indicating that the effects of genotype and water availability act independently. The genotype factor had a significant effect (*p* ≤ 0.01) on AAs, showing differences in the production of these AAs among the evaluated genetic materials. Additionally, water availability had a significant effect (*p* ≤ 0.01) on this variable, demonstrating that water deficit directly impacts amino acid synthesis in plants. No significant interactions or differences were observed for total soluble sugars (TSS) and sucrose (SUC).

**Figure 3:**
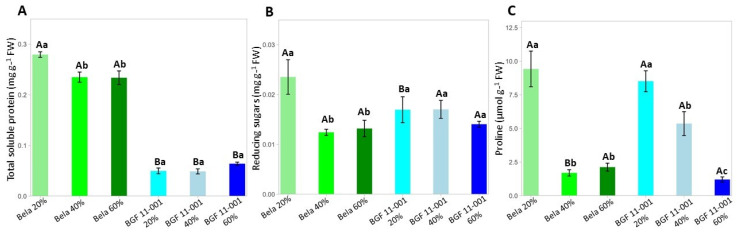
Breakdown of the interaction of Total Soluble Protein content (**A**), Reducing Sugars (**B**) and Proline (**C**) in *Stylosanthes* spp. cultivated under different water availability conditions. Means followed by different letters (uppercase for the genotype factor and lowercase for the water availability factor) differ from each other according to Tukey’s test (*p* ≤ 0.05). Error bars indicate the standard deviation of the mean.

The breakdown of the mean test for TSP content (Figure 3A) revealed distinct performances between genotypes under different water availabilities. Cv. Bela showed a significant increase in TSP content under severe water deficit (20%) compared to treatments without deficit (60%) and with moderate deficit (40%), while the BGF 11-001 accession showed no significant variation across different water availability levels. When comparing genotypes within each water condition, the cultivar remained superior to the accession at both levels. Regarding the reducing sugar content (Figure 3B), a significant increase was also observed in cv. Bela during a severe deficit (20%), responding to the imposed conditions, while BGF 11-001 again showed no significant differences between treatments. Differences between genotypes within each water availability level were observed only at 20% pot capacity, where cv. Bela had a higher mean than the accession. The proline content (Figure 3C) showed a significant difference between genotypes only at 40% water availability, with the BGF 11-001 accession demonstrating superior performance for this variable. Regarding the accumulation pattern of this molecule in response to water deficit, cv. Bela exhibited a significant increase in proline content only under 20%. In contrast, the BGF 11-001 accession already showed significant accumulation under moderate deficit (40%), with the highest content recorded at 20%.

**Figure 4:**
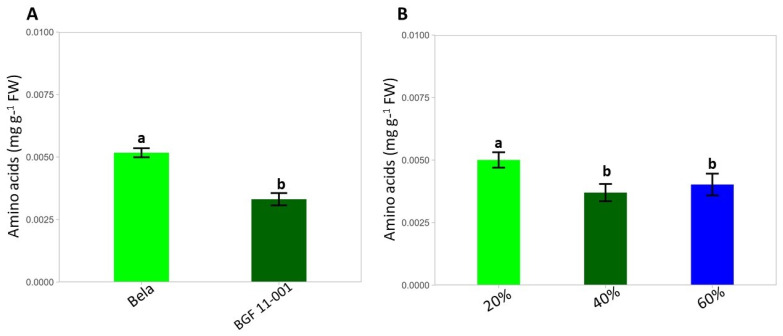
Means of the individual evaluation of genotype (**A**) and water availability (**B**) factors in the cultivation of *Stylosanthes* spp. Different letters indicate significant differences according to Tukey’s test (*p* ≤ 0.05). Error bars indicate the standard deviation of the mean.

The evaluation of the isolated effect of genotype on amino acid content (Figure 4A) indicated that cv. Bela showed greater synthesis of these AAs than BGF 11-001 accession, maintaining higher averages across treatments. Regarding the isolated effect of water availability (Figure 4B), a greater accumulation of amino acids was observed under severe water deficit conditions (20%), as this level showed significant differences compared to the other water supply levels.

**Figure 5:**
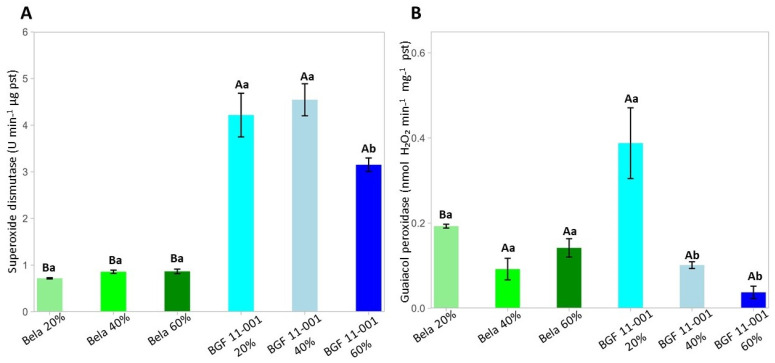
Breakdown of the interaction of Superoxide Dismutase (**A**) and Guaiacol Peroxidase (**B**) activities in *Stylosanthes* spp. grown under different water availability conditions. Means followed by different letters (uppercase for genotype factor and lowercase for water availability factor) differ from each other according to Tukey’s test (*p* ≤ 0.05). Error bars indicate the standard deviation of the mean.

A significant interaction between the factors was observed for both enzymes. The breakdown analysis of SOD (Figure 5A) revealed that the BGF 11-001 accession presented significantly higher values than cv. Bela at all water availability levels. When analyzing water availability within each genotype, it was observed that for BGF 11-001, the means obtained under 20% and 40% pot capacity were significantly higher than those recorded at 60%. In contrast, cv. Bela showed low sensitivity to water availability variation regarding the activity of this enzyme, maintaining statistically similar values across the availability levels.

Regarding the breakdown of GPX activity (Figure 5B), BGF 11-001 accession showed higher enzymatic activity than that of Bela only under the 20% water availability condition. Considering the effect of water availability within each genotype, BGF 11-001 responded to severe water deficit (20%) with a significant increase in GPX activity, while maintaining statistically similar values between the 40% and 60% levels. In turn, cv. Bela did not show significant variations among the different water availability levels.

**Figure 6:**
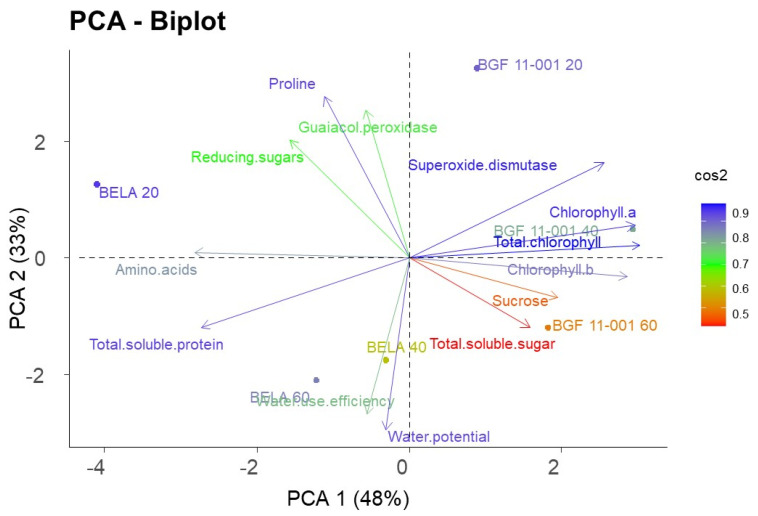
Principal component analysis (PCA) of the biochemical and physiological variables of *Stylosanthes* spp. cultivated under different water availability conditions and genotypes. The PCA was performed using mean values of all analysed variables.

The PCA biplot (Figure 6) illustrates the relationships between the treatments applied in this study and the analyzed variables. In the cultivation of *Stylosanthes* spp. under different water availability conditions, PCA explained 81% of the total variance, which was distributed across two principal components: PC1 (48%) and PC2 (33%). Superoxide dismutase activity, along with the contents of Chl*a*, Chl*b*, and Chl*tot*, showed positive correlations with each other and were associated with genotype BGF 11-001, being positively aligned with PC1. In contrast, these variables were negatively correlated with amino acid content and total soluble proteins, which, in turn, were positively associated with the Bela genotype and contributed more strongly to the negative direction of PC1.

Additionally, reducing sugar and proline contents, together with GPX activity, showed positive correlations with each other and were associated with both genotypes under severe water deficit conditions. These variables also exhibited strong positive loadings on PC2, which was the most prominent component. In contrast, water use efficiency and leaf water potential were negatively correlated with the aforementioned variables and were associated with the treatments of both Bela and BGF 11-001 under optimal water availability, as well as with the Bela genotype under moderate water deficit. These variables also contributed substantially to PC2 but with negative loadings. Sucrose and total soluble sugar content did not contribute meaningfully to the principal components represented in the biplot and, therefore, showed only weak associations with the BGF 11-001 genotype.

## 3. Discussion

Both genotypes evaluated in this study exhibited sensitivity to reduced water availability, particularly under severe deficit conditions (20%), as evidenced by the decline in water potential. Similar results were reported by Leite et al. [23], who observed a significant reduction in water potential in *Physalis angulata* L. plants subjected to 20% field capacity compared with those grown under higher water availability conditions. However, it is important to note that cv. Bela performed better at 20% water availability than the BGF 11-001 accession, according to the mean comparison test, and the PCA results further emphasized this cultivar’s greater capacity for water storage. Such differences are expected in experiments of this nature, as plant responses to stress caused by reduced water availability depend on both species and genotype [4,24].

Furthermore, the BGF 11-001 accession exhibited higher content and stability of photosynthetic pigments than the cv. Bela genotype—as evidenced by the PCA—especially regarding Chl*a* and Chl*tot*, given the reduction observed in these variables for the cultivar under severe water deficit (20%) conditions. Exposure to water stress can compromise the integrity of thylakoid membranes in chloroplasts, negatively affecting chlorophyll production [25]. Several studies, such as those by Oguz et al. [26], Guizani et al. [27], and Nour et al. [25] have also indicated that the reduction in chlorophyll levels in leaves under drought conditions can directly impact the photosynthetic efficiency of plants and the content of these pigments.

The superiority of the BGF 11-001 accession over the Bela genotype, observed for the chloroplast pigment contents quantified in this study under all water availability conditions, suggests that Bela exhibits a lower photosynthetic capacity than BGF 11-001, thereby making it more susceptible to water stress. This sensitivity can be explained by the fact that higher photosynthetic activity enhances the production of photoassimilates essential for plant survival, thereby more efficiently supporting growth and biomass production under water deficit conditions [28].

Although the BGF 11-001 accession outperformed the cv. Bela genotype, according to the mean comparison test, both showed homogeneity in Chl*b* content, with reductions observed at 20% water availability compared with the other levels. Despite the structural differences between Chl*a* and Chl*b*, the latter is predominantly located in antenna complexes, where it captures light energy and transfers it to reaction centers directly involved in photochemical reactions [29]. Interestingly, BGF 11-001 showed a reduction only in Chl*b* levels under severe water deficit (20%), which, according to the literature, is associated with an adaptive plant response to limit light energy absorption, as stomatal closure reduces the demand for this energy due to lower CO_2_ incorporation [30]. This strategy is especially relevant considering that the captured energy, instead of being used for the reducing power generated in the photochemical phase of photosynthesis, may exceed the assimilation capacity of the Calvin-Benson-Bassham cycle, promoting the accumulation of ROS and causing cellular damage [30,31].

The results for Water Use Efficiency (WUE), which were lower for both genotypes under severe water deficit (20%) than under treatments with higher water availability, align with previous findings that explain these results due to the sensitivity of net carbon assimilation under water stress conditions [32]. Under water deficit conditions, plants often close their stomata to reduce water loss through transpiration; however, this response also limits CO_2_ entry, causing a decrease in the photosynthetic rate [11].

Similar WUE performances were reported in *Pinus sylvestris* L., where WUE significantly decreased during intense drought events due to a sharper decline in photosynthesis [32]. These results were corroborated by Zhao et al. [33], who demonstrated that even species adapted to arid environments can experience reduced water use efficiency owing to the strong photosynthetic restriction imposed by low soil moisture conditions. Thus, the reduction in WUE under severe water deficit (20%), as observed in this study, can be attributed to an imbalance between CO_2_ uptake and water loss control processes, emphasizing the importance of physiological mechanisms that maintain carbon assimilation under water restriction.

Only cv. Bela varied its total soluble protein (TSP) content under different water conditions, showing a significant increase under severe deficit (20%). Despite the general tendency for decreased overall protein synthesis under water stress, the induction of specific proteins associated with water deficit response and protection against cellular damage is common [34]. Moreover, Baghery et al. [35] evaluated sesame (*Sesamum indicum* L.) genotypes with contrasting drought tolerance and observed variations in TSP content only in sensitive materials, which showed a significant increase under water deficit conditions. These findings support the results obtained for cv. Bela in this study, suggesting that TSP accumulation may be related to compensatory mechanisms in genotypes that are less adapted to water stress.

The absence of significant variation in TSS, accompanied by an increase in RS under severe water deficit (20%) was observed in cv. Bela may be related to an internal metabolic redistribution rather than an accumulation of carbohydrates. Under water stress, plants commonly maintain a relatively constant total sugar content while adjusting the profile of these compounds according to their physiological functions [36]. In this context, although sucrose is the main transport disaccharide in plants [37] and did not vary significantly, the increase in RS may indicate partial conversion of this molecule into glucose and fructose, which act as compatible osmolytes, protect cellular structures, and play roles in stress signaling [36].

This biochemical adjustment may be mediated by enzymes such as invertases and sucrose synthase, which are responsible for sucrose hydrolysis into monosaccharides for more immediate action under stress [38]. These mechanisms represent fundamental adaptive strategies in response to water deficits, as described by Farooq et al. [39]. Similar results were reported by Santos et al. [40] in *Talinum fruticosum* (L.) Juss., where a linear increase in RS content was observed in response to reduced water availability. Additionally, in *Panicum maximum* Jacq. cv. Mombaça, an increase in RS content was noted under water deficit treatment compared to the irrigated control [41].

Although genotype BGF 11-001 did not show significant changes in the levels of TSS, RS, and sucrose under water deficit conditions, a marked increase in proline content was observed, especially from the moderate water restriction level onward. This result suggests that even in the absence of variations in soluble carbohydrates, BGF 11-001 activated alternative osmoregulatory mechanisms, relying on proline synthesis as the main osmolyte to maintain the cellular osmotic potential [42]. In addition to its function as an osmotic regulator, proline stabilizes macromolecular structures and protects against reactive oxygen species [42,43,44,45]. Thus, the accumulation of this biomolecule and other amino acids may have played a compensatory role, contributing to the physiological adaptation of the genotype to low water availability.

The results reported in this study corroborate those of Baghery et al. [35] and Santos et al. [40] considering that water deficit also promoted significant increases in proline content in both studies. Moreover, in forage plants, Fariaszewska et al. [46] found increased proline synthesis compared to control treatments due to drought conditions in *Lolium perenne* L., *Lolium multiflorum* Lam., *Festuca pratensis* Huds., and *Festulolium braunii* (K. Richt).

In this context, osmotic adjustment is a mechanism that allows plants to continue the process of cell expansion even under water stress, while also contributing to the partial maintenance of stomatal opening, enabling CO_2_ assimilation during adverse conditions [47]. Therefore, the accumulation of compounds with osmotic functions helps cells manage water loss, maintain water balance, and maintain the structural integrity of membranes, which favors the tolerance of cellular structures to drought conditions [48].

Although cv. Bela showed a significantly higher mean amino acid content, also evidenced by the PCA, both genotypes demonstrated similar responses to variations in water availability, as evidenced by the increase in these biomolecules under severe water deficit conditions (20%). Amino acid accumulation is a common response in plants exposed to water stress because, together with soluble carbohydrates, these compounds can act as osmotically active agents and thus contribute to maintaining cellular water potential, allowing plants to stay hydrated and absorb water even under low soil water availability [11]. Baghery et al. [35] found similar results in *S. indicum*, noting increased amino acid accumulation under water stress, although moderate water deficit conditions (40%) were sufficient to promote this increase.

The production of ROS tends to increase proportionally to the intensity of stress, potentially causing various cellular damage such as lipid peroxidation and nucleic acid degradation [5,47]. In this context, organelles such as mitochondria, chloroplasts, and peroxisomes are among the most susceptible to the deleterious effects of excessive accumulation of these reactive molecules [7,47,49,50]. To mitigate the damage caused by excess free radicals, plants under stress conditions activate antioxidant defense systems, notably synthesizing enzymes capable of neutralizing ROS [42,47]. This mechanism is one of the main adaptive strategies used to reduce the deleterious effects of oxidative stress [47].

In this study, only BGF 11-001 showed variations in the activities of the evaluated enzymes (SOD and GPX) in response to water deficit conditions. This differential performance is consistent with observations in the literature, considering that plant responses to water stress can vary widely depending on the species and genotype involved [4,24].

The increase in SOD and GPX enzyme activities did not occur in a proportional manner. Moderate water deficit (40%) was sufficient to promote a significant increase in SOD activity, whereas the increase in GPX activity was only recorded under severe stress conditions. According to Wu and Yang [51], in winter wheat, SOD activity increases markedly under mild to moderate water deficit conditions, acting as an initial defense line by converting superoxide anions (O_2_^.−^) into hydrogen peroxide (H_2_O_2_) [49,51,52]. In contrast, enzymes such as GPX may increase only under more severe stress, possibly due to the higher demand for removing accumulated H_2_O_2_. This pattern suggests that in BGF 11-001, SOD is activated to contain the initial accumulation of ROS, whereas GPX is activated at more advanced stages, when peroxide levels reach potentially cytotoxic concentrations [51].

Furthermore, the observed correlation between proline content and GPX enzyme activity can be explained by the fact that proline accumulation has been consistently associated with enhanced antioxidant defense in plants subjected to abiotic stress [53,54]. Increased proline content is positively correlated with the activity of key antioxidant enzymes, contributing to the efficient scavenging of reactive oxygen species [54,55].

This study demonstrated distinct responses between genotypes under water deficit conditions, highlighting BGF 11-001, which showed greater accumulation of proline and amino acids under water deficit conditions and more efficient activation of the antioxidant enzymes SOD and GPX. These results indicate that BGF 11-001 possesses robust physiological defense mechanisms against oxidative stress and osmoregulation, conferring drought resilience. On the other hand, cv. Bela responded to water stress with greater accumulation of compatible solutes, suggesting a more pronounced osmotic adjustment strategy rather than activation of the antioxidant system. In the future, field studies and integration with molecular approaches are recommended to deepen the understanding of the regulatory mechanisms involved, contributing to the development of management strategies and genetic improvement aimed at water tolerance in forage legumes.

## 4. Material and Methods

### 4.1. Execution Site and Plant Material Collection

The experiment was conducted in a screenhouse at the Horto Florestal Experimental Unit of the State University of Feira de Santana (UEFS), located at coordinates 12°16′7.99″ S and 38°56′21.63″ W, at an altitude of 258 m. Two genotypes were evaluated in the experiment: accession BGF 11-001 (*Stylosanthes viscosa* (L.) Sw.), collected at coordinates 11°36′20″ S and 39°09′52.1″ W (Conceição do Coité, Bahia) and stored at BGF-UEFS; and the cultivar BRS-Bela (*Stylosanthes guianensis* (Aubl.) Sw.) (cv. Bela), developed through partnerships between Embrapa Gado de Corte and Embrapa Cerrados, Brazil.

The soil used in the experiment was collected from the 0–20 cm layer, presenting the following physicochemical attributes: pH = 6.1 in water; P = 32.0 mg L^−1^; K = 140.0 mg L^−1^; S = 11.0 mg L^−1^; Fe = 57.0 mg L^−1^; Zn = 6.2 mg L^−1^; Cu = 0.8 mg L^−1^; Mn = 13.9 mg L^−1^; B = 0.27 mg L^−1^; Ca = 2.6 mmol_c_ L^−1^; Mg = 0.7 mmol_c_ L^−1^; H + Al = 1.8 mmol_c_ L^−1^; organic matter (OM) = 2.95 g kg^−1^; base saturation (V) = 66%; sand = 660 g kg^−1^; silt = 85 g kg^−1^; clay = 288 g kg^−1^. To meet the phosphorus nutritional requirement, 0.7 g of single superphosphate was applied to each experimental unit in the study.

### 4.2. Determination of Pot Capacity

The determination of pot capacity followed the method of Bonfim-Silva et al. [56], using three pots with the same volume as those used in the experiment (8 L), filled with 8 kg of air-dried fine soil. The pots were placed in a tray filled with water to saturate the substrate by capillarity. Once the pots were completely saturated, they were removed from the trays and placed on a stand to drain excess water. The pots were weighed after 24 h of drainage using an electronic scale (precision of 0.02 g) (UPX Solution, São Paulo, Brazil). Based on this, the maximum water retention capacity of the soil was calculated, and from this value, the levels of 60%, 40%, and 20% of the pot capacity were determined.

### 4.3. Experimental Execution

For the experimental execution, a completely randomized design was employed, arranged in a 3 × 2 factorial scheme, comprising three water regimes and two genotypes, with six replicates. Seeds of both genotypes were placed in a B.O.D. (Biochemical Oxygen Demand) (Eletrolab, São Paulo, Brazil) germination chamber for 3 days, and those that showed radicle emergence were transferred to the respective pots containing substrate. Five seeds were placed in each pot.

**Figure 7:**
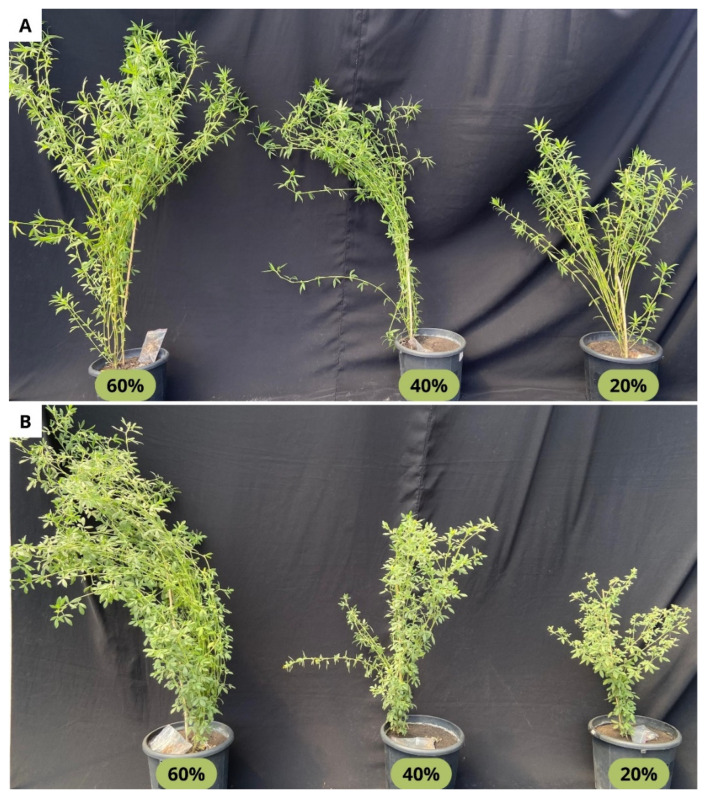
Representative photographs showing the phenotype of *Stylosanthes* spp. genotypes cultivated under different water availability conditions. Cv. Bela (**A**); BGF 11-001 (**B**). The percentage values correspond to the water availability levels applied in the experiment.

During the initial 55 days of cultivation, the plants were maintained at 60% pot capacity to allow acclimatization with daily irrigation. After this period, thinning was performed, maintaining only the most vigorous individuals in each experimental unit. Uniform cutting was performed at a height of 15 cm above the ground. Following this stage, the plants were maintained at 60% water availability for an additional 50 d before the imposition of different water levels. Subsequently, water availability was adjusted to 60%, 40%, and 20%, corresponding to the qualitative treatments of no water deficit, moderate water deficit, and severe water deficits, respectively. An electronic balance (precision of 0.02 g) was used to adjust the water availability. The pots were weighed in the morning and afternoon, and water was replenished based on the difference in weight. Cultivation was concluded after 55 days under the different water regime. The set of images shown in Figure 7 illustrates the phenotypes of the genotypes evaluated under different water availability levels applied in the study at the end of the experimental period.

**Figure 8:**
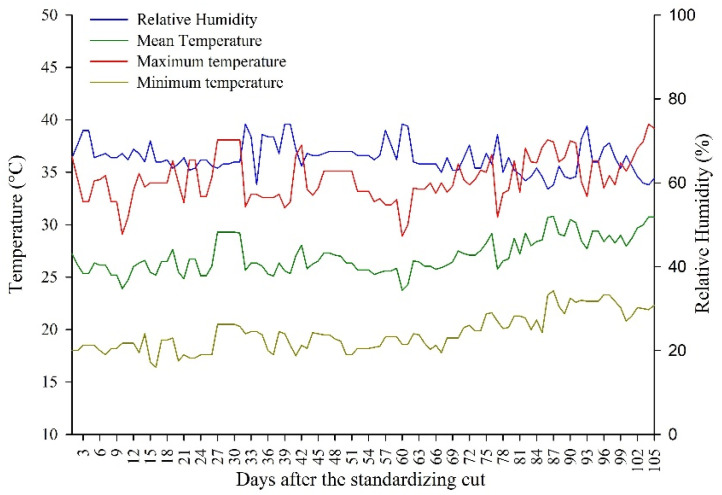
Microclimate formed inside the screenhouse.

Microclimate data were obtained using a thermo-hygrometer (EXBOM, São Paulo, Brazil) positioned inside the screenhouse. Daily observations were made, and the data from these observations are shown in Figure 8.

### 4.4. Experimental Determinations

Leaf water potential (ψw, −MPa) was determined using a Scholander pressure chamber (PMS 1000; PMS Instrument, Corvallis, OR, USA) with branches collected from the middle third during the morning period. Water use efficiency (WUE) was determined based on the ratio between productivity (g) and the amount of water applied (L) during the cultivation period, with results expressed in g L^−1^. Chlorophyll index (IFC) readings were taken using a portable chlorophyll meter (ChlorofiLOG Falker 2060, Porto Alegre, Brazil) from three fully expanded leaves located in the middle third of the plants, and the average was calculated for each experimental unit.

The extract used for biochemical analyses was obtained from 500 mg of fresh leaf tissue collected from the middle third of five individual plants. Maceration was performed separately for each sample in a porcelain mortar using 10 mL of phosphate buffer (100 mM, pH 7.0) in a porcelain mortar. The resulting extract was centrifuged at 11,000 rpm at 4 °C for 15 min, and the supernatant was collected and stored in a freezer (−18 °C).

The total soluble sugar content (TSS, mg g^−1^ FW) was determined according to Yemm and Willis [57]. In triplicate, 0.1 mL of the extract and 2 mL of anthrone reagent were added to test tubes, followed by mixing before placing the tubes in a water bath at 100 °C for 3 min. After this procedure, the tubes were allowed to cool, and the absorbance was measured using a spectrophotometer (Biospectro, Curitiba, Brazil) at 620 nm.

The content of reducing sugars (RS, mg g^−1^ FW) was determined using the dinitrosalicylic acid (DNS) method, as proposed by Miller [58]. In triplicate, 0.4 mL of the extract, 0.35 mL of distilled water, and 0.5 mL of DNS reagent were added to the test tubes. After homogenization, the tubes were placed in a water bath at 100 °C for 5 min. Subsequently, the volume was adjusted to 5 mL using distilled water, and the absorbance was measured using a spectrophotometer at 540 nm.

For sucrose quantification (SUC, mg g^−1^ FW), the difference between total soluble sugars and reducing sugars was calculated using the correction factor 0.95 [59].

The total soluble protein content was determined according to the methodology proposed by Bradford [60]. In triplicate, 0.1 mL of the extract and 5 mL of Coomassie Brilliant Blue G-250 reagent were added to the test tubes. After mixing, absorbance was measured at 595 nm using a spectrophotometer.

Amino acid content was determined according to the methodology described by Yemm and Cocking [61]. In triplicate, 0.2 mL of Extract 1, 0.8 mL of water, sodium citrate buffer (0.2 M, pH 5.0), ninhydrin (5%), and KCN (2%) in methyl cellosolve were added to test tubes. The mixture was vortexed and placed in a water bath at 100 °C for 20 min. After this period, 1.7 mL of 60% ethanol was added, the solution was vortexed again, and after cooling, the absorbance was measured using a spectrophotometer at 570 nm.

The extract used for proline quantification was prepared from 200 mg of fresh leaf tissue collected from the middle third of five individual plants. Maceration was performed in a porcelain mortar separately for each sample with the addition of 8 mL of an extraction medium composed of 3% sulfosalicylic acid. The mixture was then centrifuged at 11,000 rpm for 15 min, and the supernatant was collected and stored in a freezer. Proline quantification was performed according to the methodology described by Bates et al. [62]. To the test tubes, 0.8 mL of Extract 3, 0.2 mL of distilled water, 2 mL of the reactive solution composed of ninhydrin, phosphoric acid (6 M), and glacial acetic acid, and 2 mL of acetic acid were added. The tubes were then incubated in a water bath for 1 h. After incubation, the samples were immediately cooled in an ice bath. Subsequently, 4 mL of toluene was added to each tube and agitated to promote phase separation. The upper phase (toluene) was transferred to quartz cuvettes, and the absorbance was measured using a spectrophotometer at 520 nm.

The extract used for enzymatic activity determination was obtained from 500 mg of fresh leaf tissue collected from the middle third of five individual plants. Maceration was performed in a porcelain mortar separately for each sample with the addition of liquid nitrogen. Subsequently, 10 mL of extraction buffer was added, which consisted of 100 mM phosphate buffer (pH 7.0) and 10 mM disodium EDTA. The mixture was then centrifuged at 1500 rpm at 4 °C for 15 min, after which the supernatant was collected and stored in liquid nitrogen until analysis.

The specific activity of superoxide dismutase (SOD) was determined according to the method proposed by Giannopolitis and Ries [63], using a spectrophotometer at 560 nm, based on the difference in absorbance between illuminated and non-illuminated samples. The reaction mixture consisted of phosphate buffer (100 mM, pH 7.8), methionine (70 mM), EDTA (0.1 mM), distilled water, nitro blue tetrazolium chloride (NBT) (75 µM), and riboflavin (2 µM), which were added to a quartz cuvette containing Extract 2. The reaction was carried out under a 40 W fluorescent light source for 12 min in the dark. Simultaneously, additional cuvettes containing the same reaction mixture were kept in the dark, and absorbance readings for both illuminated and non-illuminated samples were obtained at the aforementioned wavelengths.

The specific activity of guaiacol peroxidase (GPX) was determined spectrophotometrically based on the increase in absorbance at 470 nm over 180 s, with readings taken every 15 s. The reaction was carried out in a solution containing 200 mM phosphate buffer (pH 6.5), guaiacol (100 mM), H_2_O_2_ (0.35%), and 100 μL of Extract 2. The enzymatic activity was calculated using the molar extinction coefficient of hydrogen peroxide, which was established as 26.6 mM^−1^ cm^−1^. One unit of GPX was defined as the amount of enzyme required to oxidize 1 nmol of guaiacol per minute, following the method described by Nakano and Asada [64], with adaptations from García-Limones [65].

### 4.5. Data Analysis

The assumptions necessary for analysis of variance (ANOVA) were checked using the Shapiro–Wilk test to assess the normality of residuals, while Bartlett’s test was used to verify the homoscedasticity of variances. Because of the violation of these assumptions for SOD and GPX, the data were transformed using arcsine square root transformation (arc sen√x/100). Once the assumptions were met, ANOVA was performed using a significance level of (*p* ≤ 0.05). The Tukey test (*p* ≤ 0.05) was used to evaluate the breakdown between factors and their individual effects when no significant interaction was observed between them. Additionally, principal component analysis (PCA) was performed to explore the relationships between treatments and the variables analyzed. All analyses were conducted using R statistical software (version 2024.12.0+467, R Core Team, Vienna, Austria) [66].

## 5. Conclusions

The genotype BGF 11-001 stood out for exhibiting more efficient protection mechanisms against ROS through the activation of antioxidant enzymes, in addition to the accumulation of compatible solutes, especially proline.

Cv. Bela responded with a greater accumulation of compatible solutes, suggesting a more pronounced osmotic adjustment strategy rather than antioxidant protection.

The data obtained in this study highlight the importance of the genotype × environment interaction in modulating responses to water stress and emphasize the potential of the BGF 11-001 accession as a promising material for use in environments with low water availability.

## Data Availability

The raw data supporting the conclusions of this article will be made available by the authors on request.
